# Progress in Gynæcology

**Published:** 1900-08-11

**Authors:** 


### PROGRESS IN GYNÆCOLOGY.

**The Treatment of Uterine Carcinoma.**—Dr. J. B. Deaver,[1] writing on this subject, advocates the adoption of the combined abdominal and vaginal method of hysterectomy in preference to the vaginal operation, and says he knows of no condition calling for vaginal hysterectomy which could not be better dealt with by the abdominal route. The weak spot of vaginal hysterectomy has always been the danger of injury to the ureters; though there is danger by either route, he believes there is less by the abdominal. Another disadvantage of the vaginal route is the liability of intestines to come into contact with the fingers, instruments, and ligatures; which, having traversed the vagina in cases of carcinoma, are never sterile. Vaginal hysterectomy he considers applicable only in those cases in which the carcinomatous process is strictly confined to the vaginal portion of the cervix, or the uterine canal, and when the uterus is freely movable. Dr. W. R. Pryor[2] maintains that in uterine carcinoma the surgeon should do the most radical operation possible, provided it does not carry with it too high a rate of mortality. Vaginal hysterectomy he considers suitable for only a few selected cases; his own preference is for the abdominal operation; and as every drop of blood is precious to the patients, he advocates ligation of the internal iliac arteries, which, he says, can be done with perfect safety. Picque and Manclaire[3] do not consider vaginal hysterectomy the most suitable method of dealing with cancer of the uterus. In order to remove all the disease the operation must not only be undertaken as early as possible, but must be thorough, involving the removal of all the affected lymph glands. This can only be accomplished by the abdominal route. Preliminary ligation of the uterine arteries at their point of origin affords the best method of hæmostatis. Although the mortality in the abdominal operation is four times as great as in vaginal hysterectomy, they think that if the more dangerous operation increases the chance of a radical cure, the surgeon should not hesitate to perform it in every suitable case of uterine cancer.

**The Association of Chronic Appendicitis with Disease of the Right Adnexa.**—Hiram N. Vineberg[4] calls attention to the not unfrequent association of acute catarrhal appendicitis and acute catarrhal salpingitis. Every operator who has to deal with abdominal and pelvic lesions encounters every now and then cases of marked disease of the right adnexa associated with a thickened and inflamed appendix, adherent to the pelvic mass. Such appendices usually show unmistakable signs that they have become involved secondarily to the tubal disease. Vineberg reports several cases in support of his statement. The symptoms from which these patients suffer are rather vague and indefinite; but there is one feature which stands out prominently, and that is that the subjective symptoms and general debility are out of all proportion to the physical signs found. There may be, and there usually is, indefinite tenderness over the appendicular region. On bimanual examination the right tube is found to be decidedly tender and more or less thickened, the right ovary is decidedly tender and often prolapsed. The symptoms often set in very insidiously; during the subsequent course of her ailment the patient may or may not have one or more acute attacks, which at one time may be diagnosed as appendicitis, at another right-sided salpingitis or oöphoritis, or as pelvic peritonitis. These patients are sometimes operated upon for supposed appendicitis; but after operation the pain continues, and a second laparotomy reveals a diseased right tube and ovary, which is excised and the patient cured. To avoid such a pitfall, one should adopt the rule always to investigate the condition of the right adnexa when operating for appendicitis in female patients. In the majority of cases presenting the complex phenomena described in his paper, Vineberg thinks the more desirable and prudent course to pursue is to open the abdomen in the middle line, when the appendix, uterus, and its adnexa can be treated surgically if their condition requires it. If, however, on examination prior to operation a firm adhesion of the appendix is suspected, incision through the sheath of the right rectus muscle, with traction toward the middle line, may be adopted with advantage. Of course, cases of appendicitis are sometimes met with in which neither of the two foregoing incisions will be applicable. Dr. A. McLaren calls attention to the relationship that may exist between dysmenorrhœa and appendicitis. A. J. C. Skene[5] says that the relationship explains to him certain symptoms which he had been unable to account for, *i.e.*, ovarian pain undoubtedly caused by appendicitis, the pain in the ovaries being always aggravated at the menstrual period, giving rise to the condition known as dysmenorrhœa ovarienne. In these cases the ovarian pain had disappeared when the appendix had been removed. H. A. Kelly[6] insists on the importance of examining the vermiform appendix in all cases in which the abdomen is opened, and if it shows any trace of previous inflammation it should be removed.

**The Treatment of Uterine Fibroids.**—Furneaux Jordan,[7] after discussing the pathology and symptoms of these tumours, says with regard to treatment that surgical interference is not called for in cases of small myomata not growing perceptibly, nor giving rise to pressure symptoms or severe hæmorrhage, especially if occurring near the climacteric. If, however, in spite of medical treatment the tumour increases in size, or the hæmorrhage becomes severe, something more radical must be done. Of the various surgical procedures which may be adopted he briefly reviews the three measures of myomectomy, hysterectomy, and the removal of the appendages. He says it is impossible to lay down any hard and fast rule as to which is the best method to adopt; every case must be considered on its merits, and that operation performed which we consider best for the future welfare of the patient under consideration. He does not favour the method of leaving the cervix behind, but thinks that if the case is one demanding hysterectomy, the whole uterus should be removed. As to the question of leaving one or both ovaries, he thinks this should depend to a great extent on the age of the patient; in women above the age of 38 years it is immaterial whether they are removed or left, in younger women, in whom the sudden onset of the menopause is more serious, he would favour leaving one or both ovaries. The cases he would treat by removal of the appendages are few in number, those cases in which hæmorrhage has been so severe that the patient is absolutely blanched. The shock of a hysterectomy in such cases may be just sufficient to turn the scale against recovery. Myomectomy he does not consider a satisfactory procedure, except in the case of a subperitoneal fibroid only attached to the uterus by a stalk as thick perhaps as two fingers. He cannot bring himself to regard free incision of the uterus, or the suturing up of large holes in the uterine wall, as free from the danger of hæmorrhage. When the tumour is small he would perform vaginal hysterectomy, if too large for this he recommends removal by the combined method, in which all the vaginal attachments are separated from below, Douglas pouch opened, and the bladder as far as possible separated from the uterus; then the abdomen is opened, and the broad ligament attachment dealt with from above. Howard Kelly,[8] describing the operative treatment of these tumours, recommends that we should first isolate and ligate the ovarian vessels of one side, then expose and tie the uterine vessels of the same side, next cut across the cervix and clamp the opposite uterine artery, then the round ligament, and, lastly, the ovarian vessels. Lapthorn Smith,[9] of Montreal, strongly recommends Kelly's method, and says the great advantage is, there is much less danger of injuring the ureters. He lays great stress on the importance of feeling for each individual artery and tying before cutting it, then putting a second ligature on it, as the first might be loosened after the tension of the tumour has been removed. He recommends chromicised catgut for ligaturing the vessels. He opposes leaving the ovaries and tubes, believing that sooner or later they will cause trouble. He strongly condemns vaginal morcellement on account of the risk of injury to the ureters, while complications, such as adhesion of the vermiform appendix, adhesion of intestine, and hæmorrhage, cannot be dealt with as easily as by the abdominal route. Louis Dartigues[10] reports an interesting case of an interstitial fibro-myoma in which he enucleated the tumour per vaginam, after incising the cervix bilaterally. The advantages he claims for this method are—(1) That it does not demand the sacrifice of the uterus or its appendages; (2) that the peritoneal cavity is not opened; and (3) the chances of infection are diminished. The principal points in the operation are—the ability of the surgeon to discriminate clearly the limits of the tumour, to appreciate the thickness of the uterine wall, and to avoid the risks of perforation and inversion of the uterus.

[1] Med. Rec., June 16, 1900. [2] Med. Rec., June 16, 1900. [3] Amer. Jour. of Med. Sci., Mar., 1900. [4] Med. Rec., June 2, 1900. [5] Med. Rec., June 2, 1900. [6] Post Grad., May, 1900. [7] Birmingham Med. Rev., Mar., 1900. [8] Brit. Med. Jour., June 9, 1900. [9] Brit. Med. Jour., June 9, 1900. [10] Med. Chron., May, 1900.

